# Global prevalence and antibiotic resistance profiles of bacterial pathogens in table eggs: A systematic review and meta-analysis

**DOI:** 10.14202/vetworld.2025.939-954

**Published:** 2025-04-23

**Authors:** Cyrielle Hinson, Aretas Tonouhewa, Paulin Azokpota, Georges Daube, Nicolas Korsak, Philippe Sessou

**Affiliations:** 1 Communicable Disease Research Unit, Applied Research Laboratory, Polytechnic School of Abomey-Calavi, University of Abomey-Calavi, Cotonou, Benin; 2 Department of Food Sciences, Faculty of Veterinary Medicine, FARAH-Veterinary Public Health, University of Liege, Liege, Belgium; 3 Laboratory of Food Sciences and Technology, Faculty of Agronomic Sciences, University of Abomey-Calavi, Cotonou, Benin

**Keywords:** antibiotic resistance, food safety, prevalence, *Salmonella* spp, table eggs

## Abstract

**Background and Aim:**

Eggs represent a vital dietary source globally; however, bacterial contamination poses a substantial public health risk. This systematic review and meta-analysis aimed to evaluate the global prevalence of bacterial contamination in table eggs and to characterize the antibiotic resistance profiles of these pathogens, emphasizing their implications for public health.

**Materials and Methods:**

A comprehensive bibliographic search of Web of Science, MEDLINE (PubMed), CAB Abstract, and Google Scholar databases was performed, identifying 136 studies published between 1979 and 2022. The systematic review utilized Preferred Reporting Items for Systematic Reviews and Meta-Analyses guidelines and advanced bibliometric techniques for data collection. Microsoft Excel and R software (v5.0) were employed for data consolidation and statistical analysis. Heterogeneity among studies was assessed using Higgins’ I² index, and a random-effects model was adopted for prevalence estimation and subgroup analyses.

**Results:**

Seventeen bacterial species were identified in eggs, primarily *Salmonella* spp., *Escherichia coli*, *Staphylococcus aureus*, *Campylobacter* spp., and *Listeria monocytogenes*. Overall, eggshell contamination rates exceeded those of egg contents. *Salmonella* spp. isolates exhibited complete resistance (100%) to nitrofurantoin, novobiocin, and polymyxin and substantial resistance (>50%) to commonly used antibiotics such as amoxicillin (74.5%), penicillin G (89.1%), and colistin (83.1%). *E. coli* isolates showed total resistance to penicillin G (100%) and high resistance to amoxicillin (72.2%) and ceftazidime (95.6%). Antibiotic resistance varied significantly across regions, notably higher in Asian and African isolates. Multidrug-resistant strains of *E. coli* and *Campylobacter* spp. were also identified.

**Conclusion:**

This study underscores the high global prevalence of pathogenic bacteria in poultry eggs and highlights concerning antibiotic resistance trends, particularly among *Salmonella* spp. and *E. coli*. The findings emphasize the urgent need for region-specific biosecurity protocols and antimicrobial stewardship strategies to reduce egg contamination and control antibiotic-resistant pathogens, ultimately safeguarding public health and food safety.

## INTRODUCTION

Foodborne pathogenic bacteria pose a significant global public health burden. According to estimates by the World Health Organization, approximately 600 million people around the World are affected annually by consuming contaminated food, resulting in 420,000 reported deaths due to foodborne infections or food poisoning [1]. Frequently implicated in these cases are animal-derived foods, such as meats and their derivatives, dairy products, and eggs and egg by-products [2]. Eggs and egg products hold a prominent position in the human diet because of their multifunctional properties and widespread application in the food industry, establishing them as essential dietary components [3]. They are produced on every continent inhabited by poultry, primarily by laying hens in various farming systems for human consumption.

Throughout their lifecycle, these animals are exposed to various pathogenic microorganisms such as *Salmonella* spp., *Campylobacter*, and more that can infiltrate the eggs and their derivatives through the pores on the shell, potentially serving as sources of infection or foodborne disease for consumers [4]. *Salmonella* Enteritidis, along with various other serovars, is frequently isolated from poultry and eggs [5, 6]. These bacteria, depending on the specific serovar present, can lead to gastroenteritis, typhoid fever, or paratyphoid fever, resulting in morbidity and mortality in humans [7]. In the United States, more than 2.2 million *Salmonella*-contaminated eggs are reported annually, with thousands of deaths among infected individuals [6]. Certain enteropathogenic *Escherichia coli* strains are responsible for a range of disorders, from mild diarrhea to severe conditions like hemorrhagic diarrhea, which can progress to severe kidney damage, such as hemolytic uremic syndrome [5]. Reports from various countries indicate that various enteropathogenic *E. coli* strains have been detected in table eggs, posing a substantial health risk to consumers [8]. Other pathogens such as *Listeria monocytogenes*, *Staphylococcus aureus*, and *Aliarcobacter* spp. can also contaminate eggs and egg products, potentially leading to severe foodborne infections or intoxication in consumers [9]. To better appreciate these microorganisms and prevent the spread of infections, various detection methods can be employed, depending on the type, nature, and concentration of the microorganisms. Consequently, two main approaches can be used: Direct and indirect methods. However, although more sensitive, direct methods exhibit greater execution complexity than indirect methods, which are inexpensive and quicker to implement. Since their discovery in the 20^th^ century, antibiotics have been widely used in human and veterinary medicine, notably as growth promoters and to combat pathogenic bacteria [10, 11]. Before being banned by many European countries in 2006, antibiotics were added at low doses over a prolonged period to the animals’ feed to enhance growth performance with regulatory action on the animals’ intestinal flora. However, this type of use is not permitted in animal production on most other continents [12]. Unfortunately, the frequent and consistent exposure of these microorganisms to antibiotics, combined with intrinsic and extrinsic factors, has led to the emergence of antibiotic-resistant isolates in most of these bacteria. Many of these pathogenic microorganisms have developed resistance to numerous antimicrobial agents, rendering existing antibiotic treatments ineffective and perpetuating the spread of infections [13, 14]. This increasing resistance occurs due to several causes and habits, such as the unregulated and overuse of food-grade disinfectants [15] and misuse of antibiotics for different purposes such as animal husbandry and production [16]. Exposition to increasing concentrations of food-grade disinfectants such as benzalkonium chloride, chloride dioxide, ethanol, hydrogen peroxide, and more can lead to the overexpression of multidrug efflux pump genes that contribute to the resistance of antibiotics like polymyxin B or the emergence of mutants that reduced susceptibility to aminoglycoside antibiotics [17–19]. Resistance can occur in any country and can affect anyone with no consideration of gender or age. Bacteria use several mechanisms to counteract the effects of antibiotics, including horizontal gene transfer, transformation, transduction, and conjugative plasmids [20].

Despite extensive research addressing bacterial contamination in food products, there remains a critical knowledge gap regarding the global scale of bacterial prevalence and antibiotic resistance specifically associated with table eggs. Existing studies have predominantly examined isolated regions or specific bacterial pathogens, thus providing fragmented insights that limit comprehensive understanding. In addition, most available research does not simultaneously evaluate contamination across diverse bacterial species nor systematically compare resistance profiles across different geographical regions. This lack of integrative analysis impedes the formulation of globally applicable strategies aimed at mitigating bacterial contamination risks and addressing the pressing issue of antibiotic resistance in poultry products.

To bridge this research gap, this study aims to conduct a systematic review and meta-analysis to comprehensively evaluate the global occurrence of bacterial contamination in table eggs, emphasizing pathogenic bacterial diversity and antibiotic resistance profiles. Specifically, this research seeks to (1) systematically identify and quantify the global prevalence of pathogenic bacteria in table eggs, (2) characterize antibiotic resistance patterns among the predominant bacterial isolates, and (3) examine geographical variations in contamination rates and resistance levels. Through these objectives, the study intends to inform targeted public health interventions, guide policy formulation and contribute to the advancement of global strategies for food safety and antimicrobial stewardship.

## MATERIALS AND METHODS

### Ethical approval

This study did not require an ethical approval as it was based on information/data retrieved from published studies already available in the public domain. The systematic review followed the PRISMA 2020 guidelines. The completed checklist is available as supplementary file.

### Study period and location

The screening of relevant studies, as well as the compilation and analysis of data, was conducted from April 2023 to January 2024 at the University of Abomey-Calavi in the Republic of Benin and the University of Liege in Belgium.

### Study scope

This systematic review followed the PICOS framework and targeted the population of table eggs from laying hens and their associated bacterial contaminants. The intervention or exposure studied was the presence of bacterial contamination and/or antibiotic resistance in these eggs, without any human intervention. A direct comparison of detection rates between different egg matrices was done. Expected primary outcomes were the prevalence of bacterial contamination (as a percentage of contaminated eggs), while secondary outcomes included antibiotic resistance rates (as a percentage of resistant isolates), bacterial species diversity, and geographic distribution. The study design included original full-text studies reporting natural bacterial contamination in table eggs. Experimental studies with artificial contamination or inhibitory treatments were excluded.

### Search strategy

To compile a comprehensive global database of research endeavors dedicated to the microorganism examination of contamination in table eggs, a novel application of the Preferred Reporting Items for System-atic Reviews and Meta-Analyses (PRISMA) protocol was employed, incorporating advanced bibliometric techniques to enhance the robustness of the systematic review. Four bibliographic databases, namely Web of Science, MEDLINE (PubMed), CAB Abstract, and Google Scholar, were used using the following keywords: “(“prevalence” OR “contamination” OR “occurrence” AND “drug resistance” OR “antibioresistance” OR “antibiotic resistance” OR “antimicrobial resistance” AND “bacterial” OR “microbial” OR “microbium” OR “pathogen” AND “egg”)” were scoured. The search was limited to peer-reviewed articles published in English or French between 1979 and 2022. Three investigators studied the titles and abstracts of all the articles and retrieved data.

### Eligibility criteria

**Figure 1:**
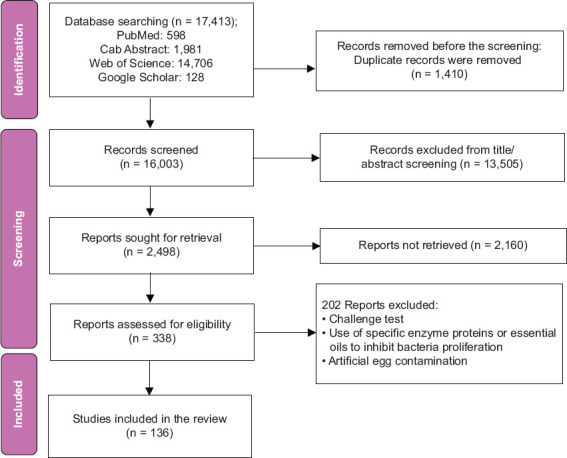
Flow chart displaying the results of the literature search.

After identifying various articles using the specified keywords in the previously mentioned data-bases, inclusion and exclusion criteria were carefully established to classify the most pertinent studies based on their titles and abstracts. The PRISMA flow chart (Figure 1) was used to retrieve relevant studies with the following inclusion and exclusion criteria.

### Inclusion criteria

Original studies, available in full text, reporting the presence of potentially pathogenic bacteria in eggs from laying hens exclusively (shells, yolk, or albumen). Studies that provide, within the full text, comprehensive information on the total sample size studied, the number of positive samples, the prevalence rate of bacteria, the methods of diagnosis used, such as culture non-selective or selective enrichment culture/process, serotyping, and polymerase chain reaction (PCR) for the detection of the different microorganisms.

### Exclusion criteria

Experimental studies involving artificial egg contamination, challenge tests, or the use of specific enzymes, proteins, or essential oils to inhibit the proli-feration of pathogenic bacteria were excluded based on the studies’ titles and abstracts.

### Data collection

All eligible articles were examined according to their full text. The data were carefully extracted and recorded in an Excel spreadsheet, and the information was classified as follows: The first author’s name, year of publication, sampling period, study area, sample size, type of product (eggshell or contents), bacterial identification method, technique for detecting anti-biotic resistance, count of eggs tested positive for distinct bacteria, and the number of antibiotic-resistant bacterial isolates found in these eggs.The characteristics of the included studies are summarized in the supplementary table.

### Statistical analysis

All the gathered data were consolidated into a Microsoft Excel database, and statistical analyses were performed using R software version 5.0, employing the meta and metafor packages. To evaluate heterogeneity among the included studies, Higgins’ I^2^ index was used. I^2^ values exceeding 50% indicated significant variability among the studies, whereas values below 50% indicated lower heterogeneity. In cases in which analyses revealed high heterogeneity within the studies, a random-effects model was employed for the meta-analysis to calculate the overall prevalence of pathogenic bacteria in eggs and antibiotic resistance. The pooled prevalence of eggs extracted from the different studies was used to obtain the overall prevalence. For the subgroup analysis, the detection rate was based on the matrices analyzed, namely eggshell and egg contents, which included the yolk and albumen. Excel-365 was used to plot a map showing the spatial distribution of all studies and to step up a heatmap showing antibiotic resistance of *Salmonella* spp., *E. coli*, *Campylobacter* spp., and *S. aureus* isolates from table eggs. The percentages pre-sented in the heatmap were derived by averaging the percentages of bacteria resistant to each antibiotic over the total number of bacteria tested in each study and across different countries.

## RESULTS

### Characteristics of eligible studies

**Figure 2:**
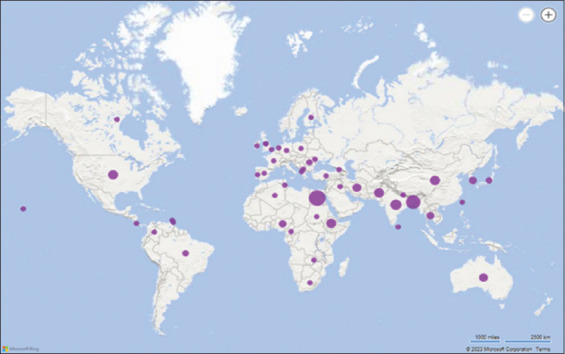
Spatial distribution of eligible studies. *The purple dots indicate the countries of the studies included in the meta-analysis.

A total of 17,413 scientific publications were identified across the four databases under investigation. After eliminating duplicates and irrelevant research, 2498 papers were evaluated based on their titles and abstracts. This led to the exclusion of 2160 studies, leaving 338 articles that met the predefined inclusion and exclusion criteria. After an in-depth review of the full text of the 338 articles, 136 studies were ultimately selected for inclusion based on the inclusion criteria mentioned earlier. In summary, all studies included in the synthesis were published between 1979 and 2022. These studies collectively involved the analysis of 217,995 chicken egg samples collected from 47 countries spanning all four continents. A notable percentage of these investigations (43.3%) were conducted in Asia, followed by Africa (24%), Europe (15.43%), America (12.5%), and Oceania (6%) as shown in Figure 2. The study identified a wide range in the number of eggs examined, ranging from 15 to 105,033. The different parts of the eggs used as matrices for detecting bacteria included the shells, yolk, albumen, or the entire contents (a mixture of yolk and albumen). In some of these articles, antibiotic resistance in the isolated bacteria was assessed using the disk diffusion method or broth microdilution method for four specific bacteria: *Salmonella* spp., *E. coli*, *S. aureus*, and *Campylobacter* spp.

### Taxonomy diversity and occurrence of bacterial communities associated with table eggs

Considering the entire set of eligible studies (136), bacteria associated with table eggs on a global scale encompassed 17 bacterial genera/species separated into two groups: *Salmonella* spp., *Campylobacter* spp., *S. aureus*, *L. monocytogenes*, *Yersinia* spp., *Arcobacter*, *Shigella*, and *Vibrio* for pathogenic bacteria and *Aeromonas*, *Proteus mirabilis*, *Klebsiella*, *Citrobacter*, *Enterobacter*, *Hafnia*, *Pantoea*, *Serratia*, *E. coli* for non- or potentially-pathogenic bacteria (Table 1). As illustrated in Table 1, *Salmonella* was identified in most of the studies included in the synthesis (n = 96). Following the diagnosis of *Salmonella*, *E. coli* was detected in 40 studies, while *S. aureus*, *Campylobacter*, and *Listeria* were observed in 23, 15, and 9 studies, respectively. Within the scope of comprehensive analysis, global detection rates exhibit variations from one microorganism to another due to the use of different detection methods. These diverse methods rely on the use of selective media and/or enrichment broths and PCR techniques, which can influence the varied rates obtained. *S. aureus* emerged as the most prevalent microorganism (p = 34.3%; 95% confidence interval [CI]: 19.7–50.5), with a presence both on eggshells (35.5%) and within the egg itself (19.1%). Subsequently, *E. coli* isolates were identified at a frequency of 18.5% (p = 18.5%; 95% CI: 12.6–25). Furthermore, a lower incidence, below the 10% threshold, was noted for *Salmonella* spp. (p = 7.1%; 95% CI: 05.18–9.37), while the isolation of *Hafnia alvei* was infrequent, with a detection rate below 1% in the eggs subjected to analysis for its presence (p = 0.8%; 95% CI: 0.1–1.8). Other bacteria, including *P. mirabilis*, *Klebsiella* spp., *Yersinia* spp., *Aeromonas* spp., *Arcobacter* spp., *Citrobacter* spp., *Enterobacter* spp., *Hafnia* spp., *Pantoea* spp., *Serratia* spp., *Shigella* spp., and *Vibrio* spp., have also been identified, but only in a limited number of studies. Regarding the diversity observed among the Salmonella isolates, a wide range of variants were identified, with 54 serovars (Africana, Agona, Albany, Amsterdam, Anatum, Bareilly, Blockley, Bongori, Braenderup, Caracas, Cerro, Corvallis, Derby, Dublin, Emek, Enteritidis, Gallinarum, Georgia, Give, Group C, Hadar, Heidelberg, II, Indiana, Infantis, Javiana, Kentucky, Lagos, Livingstone, Mbandaka, Maleagridis, Mo-lade, Montevideo, Norwich, Ohio, Panama, Paratyphi, Paratyphi A, Pullorum, Richmond, Rissen, Rough, Senftenberg, Singapore, Tennessee, Thompson, Typhi, Typhimurium, Virchow, Weltevreden, Worthington, 4.12:d:-, 1.6, 7: i, V:-, 1.6, 7: ZIO:-). However, the most frequently encountered serovars were Enteritidis (64.9%), followed by Typhimurium (8%), Cerro (3.3%), Infantis (1.9%), and Mbandaka (1.6%). In contrast, lower diversity was observed for *E. coli*, with 13 distinct serovars represented by the following variants: O157:H7, O44:K74, O158:K, O119:K67, O119:K69, O25:K11, O86:K61, O128:K67, O126:K71, O127:K63, O125:K70, O114:K90, and O111:K58.

**Table 1:** Frequency of bacteria isolated from table eggs.

Bacteria	Product	Prevalence (%)	95% CI	I^2^	Number of studies
*Aeromonas* spp.	Eggshell	-	-	-	-
	Egg content	-	-	-	-
	Overall	1.5	0.6–2.6	98.1	2
*Arcobacter* spp.	Eggshell	-	-	-	
	Egg content	-	-	-	-
	Overall	0.8	0.2–1.9	98.1	1
*Campylobacter* spp.	Eggshell	11.1	1.4–27.8	97.6	9
	Egg content	8.8	0.0–28.6	97.6	3
	Overall	8.0	2.0–17.3	98.1	12
*Citrobacter* spp.	Eggshell	7.4	0.0–25.2	77.7	2
	Egg content	8.0	0.2–23.1	77.7	2
	Overall	7.8	2.0–16.4	98.1	3
*Enterobacter* spp.	Eggshell	5.7	3.32–8.7	86.8	3
	Egg content	14.5	1.9–35.0	86.8	3
	Overall	9.9	3.7–18.4	98.1	6
*Escherichia coli*	Eggshell	25	16.6–34.4	97.1	24
	Egg content	7.9	3.3–14.2	97.1	8
	Overall	18.5	12.7–25.0	98.1	30
*Hafnia alvei 1*	Eggshell	-	-	-	-
	Egg content	-	-	-	-
	Overall	0.8	0.17–1.81	98.1	1
*Klebsiella* spp.	Eggshell	40	26.11–54.8	94	1
	Egg content	7.2	2.2–14.6	94	1
	Overall	13.6	2.5–30.9	98.1	3
*Listeria* spp.	Eggshell	17.0	0.0–50.5	95.7	4
	Egg content	17.3	4.3–36.0	95.7	5
	Overall	17.1	5.4–33.2	98.1	7
*Pantoea* spp.	Eggshell	-	-	-	-
	Egg content	-	-	-	-
	Overall	2.8	1.5–4.4	98.1	1
*Proteus mirabilis*	Eggshell	-	-	-	-
	Egg content	-	-	-	-
	Overall	12.3	2.1–28.5	98.1	3
*Salmonella* spp.	Eggshell	8.5	5.5–12.2	99.1	70
	Egg content	6.0	3.4–9.3	99.1	43
	Overall	7.1	5.2–9.4	97.4	96
*Serratia* spp.	Eggshell	-	-	-	-
	Egg content	3.5	0.0–14.3	95.1	1
	Overall	3.5	0.0–14.3	98.1	3
*Shigella* spp.	Eggshell	-	-	-	-
	Egg content	-	-	-	-
	Overall	4.7	1.9–8.4	98.1	1
*Staphylococcus aureus*	Eggshell	35.5	14.9–59.3	98.9	12
	Egg content	19.1	9.2–31.5	98.9	8
	Overall	34.3	19.7–50.6	98.1	17
*Vibrio* spp.	Eggshell	-	-	-	-
	Egg content	-	-	-	-
	Overall	1.7	0.2–4.4	98.1	1
*Yersinia* spp.	Eggshell	0.7	0.0–2.0	0	1
	Egg content	1.0	0.3–2.2	0	2
	Overall	0.9	0.3–1.7	98.1	2

CI=Confidence interval; I^2^=Heterogeneity

### Subgroup analysis

To compare the variation in detection rates of different microorganisms based on the matrices used for microbiological analyses, a subgroup analysis was conducted. The results are presented in Table 1. For all microorganisms, a higher level of contamination was observed in the eggshells compared to the egg contents. However, since the content of eggs is sterile after laying, the presence of microorganisms at various levels in the content may result from challenges in achieving sterile collection of egg content, insufficient disinfection of the shell, handling and storage conditions, or the diagnosis methods used. *S. aureus* was detected in 12 studies with a frequency of 35.5% (95% CI: 14.8–59.2) on eggshells, in contrast to 19.1% (95% CI: 9.1–31.4) in the egg contents. *E. coli* was found on eggshells at a rate of 25% (95% CI: 16.5–34.4), which was higher than the rate observed in egg contents, at 7.9% (95% CI: 3.2–14.2). For *Salmonella* spp., the contamination rate on eggshells was 8.5% (95% CI: 5.4–12.2), slightly higher than the rate observed for egg contents, which was 6.0% (95% CI: 3.3–9.2). In addition, *Campylobacter* spp. were detected on eggshells with a frequency of 11.1% (95% CI: 1.3–27.7), compared to 8.8% (95% CI: 0.0–28.6) in the egg contents.

### Antibiotic resistance of isolates

**Figure 3:**
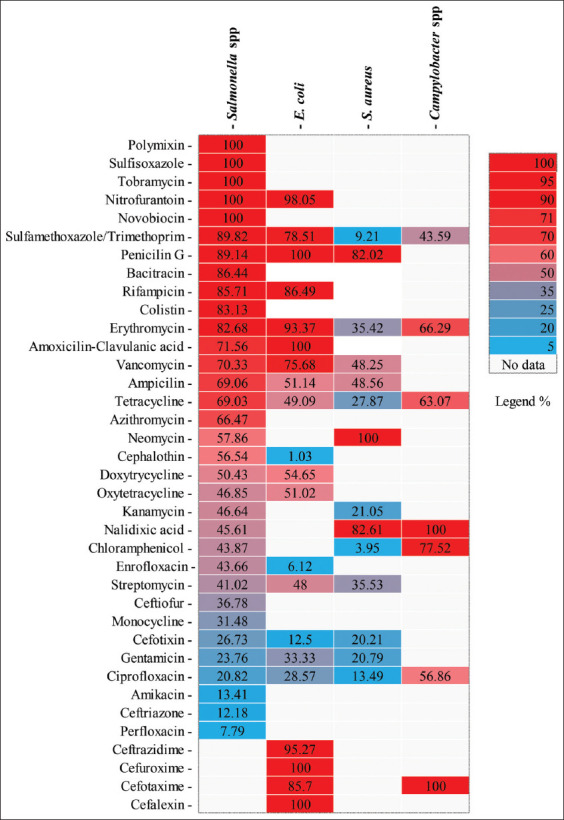
Heatmap showing the antibiotic resistance of *Salmonella* spp. *Escherichia coli*, *Campylobacter* spp., and *Staphylococcus aureus* isolated from table eggs. *The percentages presented in the heatmap were calculated by averaging the percentages of bacteria resistant to each antibiotic over the total number of bacteria tested in each study and per country.

The heatmap of antibiotic resistance among the four bacteria commonly isolated from table eggs is illustrated in Figure 3.

#### Salmonella spp.

*Salmonella* isolates exhibit the highest rates of resistance to antibiotics used for their control in both human and veterinary medicine. An analysis of the overall calculated resistance rates (Table 2) indicated that *Salmonella* spp. isolates from eggs developed complete resistance to three antibiotics: Nitrofurantoin at 100% (95% CI: 87.3–100), novobiocin at 100% (95% CI: 97.1–100), and polymyxin at 100% (95% CI: 97.1–100). These results were derived from two studies on each antibiotic. Furthermore, a very high level of resistance (p > 50%) was observed to 15 antibiotics, including colistin 83.1% (95% CI: 20.8 – 100), cephalothin 56.5% (95% CI: 23.7–86.9), amoxicillin 74.5% (52.1–92.2), amoxicillin-clavulanic acid 71.6% (95% CI: 59.3–82.6), penicillin G 89.1% (95% CI: 49–100), vancomycin 70.3% (95% CI: 3.4–100), bacitracin 86.4% (95% CI: 36–100), tetracycline 69% (95% CI: 46–88.6), erythromycin 82.7% (95% CI: 56.6–99.2), azithromycin 66.5% (95% CI: 0.3–100), ampicillin 69.1% (95% CI: 46.5–88), neomycin 57.9% (95% CI: 0.0–100), sulfamethoxazole/trimethoprim 89.8% (95% CI: 58–100), and doxycycline 50.4% (95% CI: 19.3–81.4). Resistance to antibiotics, such as rifampicin, 85.7% (95% CI: 48.3–100); enrofloxacin, 43.7% (95% CI: 32.3–55.4); sulfamethoxazole, 100% (95% CI: 97.2–100); and minocycline, 31.5% (95% CI: 19.7–44.6) were reported only in one study. However, only three antibiotics remained effective against most tested Salmonella isolates (p < 20%): Pefloxacin (7.8%), ceftriaxone (12.2%), and amikacin (13%).

**Table 2:** Antibiotic resistance of *Salmonella* spp. isolates.

Family	Antibiotic	Prevalence (%)	95% CI	I^2^ (%)	N	n	Number of studies
Aminoglycosides	Amikacin	13.4	7.3–20.9	0.0	103	14	2
	Gentamicin	23.8	10.6–39.7	85.5	258	61	8
	Kanamycin	46.6	33.7–59.8	75	256	128	7
	Neomycin	57.7	0.0–100	99.0	63	40	2
	Streptomycin	41.0	26.1–56.7	75.9	224	83	7
	Tobramycin	100	86.7–100	0.0	9	9	2
Rifamycin	Rifampicin	85.2	48.3–100	-	7	6	1
Beta-lactams	Amoxicillin	74.5	52.1–92.2	91.2	184	132	8
	Amoxicillin-Clavulanic acid	71.6	59.3–82.6	31.1	121	86	4
	Ampicillin	69.1	46.9–87.9	94.7	435	272	15
	Cefoxitin	26.7	2.6–61.8	92.4	116	13	3
	Ceftiofur	36.8	13.6–63.3	86.1	111	42	3
	Ceftriaxone	12.2	1.2–30.1	82.2	116	18	3
	Cephalothin	56.5	23.7–86.9	70.1	57	33	3
	Penicillin G	89.1	49–100	93.4	80	44	4
Cyclins	Doxycycline	50.4	19.3–81.4	58.7	34	19	2
	Minocycline	31.5	19.7–44.6	-	54	17	1
	Oxytetracyclines	46.9	30.0–64.0	50.0	69	33	2
	Tetracycline	69.0	46.0–88.6	90.6	349	236	11
Furans	Nitrofurantoin	100	87.4–100	0.0	15	15	2
Glycopeptides	Vancomycin	70.3	3.4–100	92.8	49	42	2
Macrolides	Azithromycin	66.5	0.3–100	81.3	58	9	4
	Erythromycin	82.7	56.6–99.15	94.9	234	158	10
Non-classified	Novobiocin	100	97.1–100	0.00	56	56	2
Phenicols	Chloramphenicol	43.9	26.4–62	79.1	178	89	8
Polypeptides	Polymyxin	100	97.1–100	0.0	66	66	2
	Colistin	83.1	20.7–100	97.2	98	64	2
	Bacitracin	86.4	36–100	90.6	41	31	2
Quinolones and fluoroquinolones	Ciprofloxacin	20.8	7.6–37.2	85.1	301	77	9
	Enrofloxacin	43.7	32.3–55.4	-	71	31	1
	Nalidixic acid	45.6	23.8–68.1	88.1	279	125	10
	Pefloxacin	7.8	3.0–14.8	0.0	98	8	2
Sulfamides	Sulfisoxazole	100	97.2–100	-	61	61	1
	Sulfamethoxazole/Trimethoprim	89.8	58.0–100	61.3	70	51	3
	Sulfamethoxazole	23.3	0.0–84.1	96.3	66	16	2
	Trimethoprim	39.1	0.0–100	66.0	28	6	2

CI=Confidence interval, I^2^=Heterogeneity, n=Number of resistant isolates; N=Number of tested isolates

The variation in resistance to 10 antibiotics among *Salmonella* spp. isolates across continents is presented in Table 3. Subgroup analysis revealed a significant distribution (p < 0.05) of prevalence based on regions for three antibiotics, namely ampicillin, gentamicin, and streptomycin, unlike other antimicrobials for which observed variations were not significant (p > 0.05). For ampicillin, the lowest resistance levels were observed in Oceania (4.9%) compared with Asia (80.6%), Africa (79.3%), and Europe (59.3%); p < 0.001. In contrast, for gentamicin, the prevalence of antibiotic resistance was higher in Europe (61%) and Africa (57%) compared with the Asian continent (23%), where the resistance level to this antibiotic was lower; p < 0.005. A similar situation was observed for streptomycin, where *Salmonella* spp. isolated from eggs in Europe and Africa were highly resistant (71% and 63%, respectively), whereas in Asia, the phenomenon appeared to be moderate, with 75% of *Salmonella* spp. isolates still sensitive (p < 0.05).

**Table 3:** Antibiotic resistance of *Salmonella* spp. isolates from different continents.

Antibiotics	Region	Prevalence (%)	95% CI	Number of studies	I^2^	Subgroup analysis (random effect model)
Amoxicillin	Africa	68.8	36.2–94.6	4	89.6	p = 0.5477
	Asia	80.8	46.7–99.9	5		
	Total	76.6	51.8–94.7	9		
Ampicillin	Africa	80.6	43.8–100	4	93.5	p = 0.0001
	Asia	79.3	55.1–96.7	10		
	Europe	59.3	42.4–75.5	3		
	Oceania	4.9	0.8–14.1	1		
	Total	72.7	50.6–90.9	18		
Chloramphenicol	Africa	61.7	0.0–100	2	69.3	p = 0.5498
	Asia	38.2	14.2–65.3	5		
	Europe	55.5	38.9–65.3	2		
	Total	44.8	28.3–61.8	9		
Ciprofloxacin	Africa	28.6	1.0–68.2	1	83.7	p = 0.2595
	Asia	22.4	9.9–37.8	8		
	Europe	100	0.0–100	1		
	Total	22.4	9.1–38.4	10		
Erythromycin	Africa	62.5	25.9–93.3	1	93.2	p = 0.3062
	America	100	50.0–100	1		
	Asia	91.5	61.7–100	11		
	Total	90.6	63.7–100	13		
Gentamicin	Africa	57.1	18.6–91.9	1	86.2	p = 0.0041
	Asia	23.0	9.6–39.4	8		
	Europe	61	45.5–75.5	1		
	Total	31	14.6–49.8	10		
Kanamycin	Africa	28.6	(1.0–68.2)	1	77.5	p = 0.2826
	Asia	51,1	(33.3–68.8)	6		
	Europe	61	(45.5–75.5)	1		
	Total	50.6	36.1–65	8		
Nalidixic acid	America	41.7	(0.0–100)	2	87.8	p = 0.8124
	Asia	62.2	(31.8–88.9)	7		
	Europe	69.8	(48.9–88)	2		
	Total	60.1	35.5–82.9	11		
Streptomycin	Asia	34.7	(19.7–51.2)	5	80.3	p = 0.166
	Europe	63.4	(48–77.6)	1		
	Africa	71.4	(43.3–93.7)	2		
	Total	47.1	30.3–64.2	8		
Tetracycline	Africa	71	23–100	3	90.5	p = 0.5527
	Asia	75.6	52.8–92.9	6		
	Europe	44.1	0.0–98.5	3		
	Total	69.5	49.5–86.8	12		

CI=Confidence interval, I^2^=Heterogeneity. The values in bold correspond to the combined overall prevalence, the 95% confidence interval and the number of antibiotic resistance studies for all the regions listed, for each antibiotic.

#### Escherichia coli

Regarding the resistance profiles of the studied *E. coli* isolates in various articles (Table 4), complete resistance was observed to penicillin G 100% (95% CI: 96.7–100). In addition, a very high level of resistance (p > 50%) was noted for amoxicillin 72.2% (95% CI: 20.6–100), ceftazidime 95.6% (95% CI: 82.2–100), doxycycline 54.7% (95% CI: 43.9–65.2), ampicillin 51.1% (95% CI: 11.2–90.3), sulfamethoxazole/trimethoprim 78.5% (95% CI: 6.8–100), erythromycin 93.4% (95% CI: 7.6–100), and nitrofurantoin 98.1% (95% CI: 72.4–100). However, total resistance to several antibiotics, such as oxacillin 100% (95% CI: 40–100), cefuroxime 100% (95% CI: 76.8–100), cephalexin 100% (95% CI: 92.9–100), and amoxicillin-clavulanic acid 100% (95% CI: 76.8–100), and high-level resistance (p > 50%): vancomycin 75.7% (95% CI: 60.4–88.3), cefotaxime 85.7% (95% CI: 70.7–98.3), oxytetracycline 51% (95% CI: 37–65.0), and rifampicin 86.5% (95% CI: 73.3–95.9), were reported in one study at each time. Moreover, most *E. coli* isolates remained sensitive to norfloxacin 15.2% (95% CI: 0.2–43.4), ciprofloxacin 28.6% (95% CI: 1.0–68.2), enrofloxacin 6.1% (95% CI: 0.8–14.9), cefotaxime 12.5% (95% CI: 1.7–29.3), and cephalexin 1.0% (95% CI: 0.0–3.1).

**Table 4:** Antibiotic resistance of *Escherichia coli* isolates.

Family	Antibiotic	Prevalence (%)	95% CI	I^2^	N	n	Number of studies
Aminoglycosides	Gentamicin	33.3	15.6–53.6	-	24	8	1
	Streptomycin	48	2.2–96.3	99.2	322	100	3
Rifamycin	Rifampicin	86.5	73.3–96	-	37	32	1
Beta-lactams	Amoxycillin	72.2	20.6–100	95.9	100	55	3
	Amoxicillin-Clavulanic acid	100	76.8–100	-	7	7	1
	Ampicillin	51.1	11.2–90.3	96.5	218	94	5
	Cephalexin	100	93–100	-	24	24	1
	Cefotaxime	85.7	70.7–98.27	-	24	21	1
	Cefoxitin	12.5	1.7–29.28	-	24	3	1
	Ceftazidime	95.3	82.8–100	0	31	29	2
	Cefuroxime	100	76.8–100	-	7	7	1
	Cephalothin	1.0	0.0–3.1	-	194	2	1
	Oxacillin	100	95.4–100	-	37	37	1
	Penicillin G	100	96.7–100	0	51	51	2
Cyclines	Tetracycline	49.1	20.1–78.4	96.8	401	146	5
	Oxytetracycline	51.0	37–65.0	-	49	25	1
	Doxycycline	54.7	43.9–65.2	0	86	46	2
Furans	Nitrofurantoin	98.1	72.4–100	69.3	44	43	2
Glycopeptides	Vancomycin	75.7	60.4–88.3	-	37	28	1
Macrolide	Erythromycin	93.4	70.6–100	72.6	51	45	2
Multidrug resistance	Amoxicillin-clavulanic-Ampicillin- tetracycline-sulfamethoxazole-trimethoprim	5.5	0.7–13.4	-	55	3	1
	Ampicillin-Amoxicillin-clavulanic	16.4	7.6–27.5	-	55	9	1
	Ampicillin-Amoxycyline-clavulanic- Tetracycline- sulfamethoxazole- trimethoprim-Gentamicin	1.8	0.0–7.6	-	55	1	1
	Ampicillin-Tetracycline	7.3	1.6–15.9	-	55	4	1
	Ampicillin-Tetracycline- Sulfamethoxazole-Trimethoprim	1.8	0.0–7.6	-	55	1	1
	Ampicillin-Tetracycline- Sulfamethoxazole- Trimethoprim-Gentamicin	1.8	0.0–7.6	-	55	1	1
	Cephalothin-tetracycline	0.5	0.0–2.2	-	194	1	1
	Gentamicin-streptomycin-sulfamethoxazole	1.0	0.0–3.1	-	194	2	1
	Gentamicin-Tetracycline	2.1	0.4–4.6	-	194	4	1
	Streptomycin-Tetracycline	2.6	0.7–5.4	-	194	5	1
Quinolones	Ciprofloxacin	28.6	1.0–68.2	-	7	2	1
	Enrofloxacin	6.1	0.8–14.9	-	49	3	1
	Norfloxacin	15.2	0.2–43.4	84.4	73	10	2
	Ofloxacin	28.6	1.0–68.2	-	7	2	1
Sulfamides	Sulfamethoxazole/Trimethoprim	78.5	6.8–100	98.2	86	56	2

CI=Confidence Interval, I^2^=Heterogeneity, n=Number of resistant isolates, N=Number of tested isolates

#### S. aureus

Table 5 presents the rate of antibiotic resistance in *S. aureus*. More than 60% of the isolates exhibited resistance to amoxicillin at 74.3% (95% CI: 34.7–99.1), oxacillin at 62.8% (95% CI: 2.7–100), and penicillin G at 82% (95% CI: 55.1–99.4). In addition, moderate resistance (0.20 < p < 0.50) was observed with cefoxitin at 20.2% (95% CI: 4.1–43.9), vancomycin at 48.3% (95% CI: 0.0–100), ampicillin at 48.6% (0.0–100), gentamicin at 20.8% (95% CI: 3.4–46.6), erythromycin at 35.4% (95% CI: 7.1–70.7), and tetracycline at 27.9% (95% CI: 14.4–43.6). However, resistance to several antibiotics was observed, although each was only reported in one study: Kanamycin 21.1% (95% CI:12.5–31), streptomycin 35.5% (95% CI: 25.1–46.7), sulfamethoxazole 36.4% (95% CI: 17.3–57.8), neomycin 100% (95% CI: 76.8–100), levofloxacin 36.9% (95% CI: 28.2–46.2), moxifloxacin 21.6% (95% CI: 14.4–29.8), and nalidixic acid 82,6% (95% CI: 64–95.9). Conversely, most *S. aureus* isolates remained sensitive (p < 20%) to several antibiotics, including daptomycin 3.6% (95% CI: 0.8-8.0), chloramphenicol 4% (95% CI: 0.5–9.8), tigecycline 11.7% (95% CI: 6.3–18.4), ciprofloxacin 13.5% (95% CI: 2.7–29.7), gatifloxacin 7.2% (95% CI: 3.0–12.9), and linezolid 5.5% (95% CI: 1.2–12.1).

**Table 5:** Antibiotic resistance of *Staphylococcus aureus* isolates.

Family	Antibiotic	Prevalence (%)	95% CI	I^2^	N	n	Number of studies
Aminoglycosides	Gentamicin	20.8	3.4–46.6	93.6	210	47	3
	Neomycin	100	76.8–100	-	7	7	1
	Kanamycin	21.1	12.5–31	-	76	16	1
	Streptomycin	35.5	25.1–46.7	-	76	27	1
Beta-lactams	Amoxicillin	74.3	34.7–99.1	91.6	99	63	2
	Ampicillin	48.6	0.0–100	99.4	187	78	2
	Cefoxitin	20.2	4.1–43.9	91.8	187	43	2
	Daptomycin	3.6	0.8–8.1	-	111	4	1
	Oxacillin	62.8	2.7–100	96.7	61	41	2
	Penicillin G	82	55.1–99.4	94	233	155	5
Cyclines	Tetracyclines	27.9	14.4–43.6	81	210	53	3
	Tigecycline	11.7	6.3–18.4	-	111	13	1
Glycopeptides	Vancomycin	48.25	0.0–100	90.5	30	10	2
Macrolides	Erythromycin	35.4	7.1–70.7	97.1	210	65	4
Multidrug resistance	Quinupristin-Dalfopristin	1.8	0.0–5.4	-	111	2	1
Oxazolidinones	Linezolid	5.5	1.2–12.1	-	73	4	1
Phenicols	Chloramphenicol	4	0.5–9.8	-	76	3	1
Quinolones	Ciprofloxacin	13.5	2.7–29.7	82.4	232	23	4
	Gatifloxacin	7.2	3.0–12.9	-	111	8	1
	Levofloxacin	36.9	28.2–46.2	-	111	41	1
	Moxifloxacin	21.6	14.4–29.8	-	111	24	1
	Nalidixic acid	82.6	64–95.9	-	23	19	1
Sulfamides	Sulfamethoxazole	36.4	17.3–57.8	-	22	8	1
	Sulfamethoxazole/Trimethoprim	9.2	3.6–16.9	-	76	7	1

CI=Confidence Interval, I^2^=Heterogeneity, n=Number of resistant isolates, N=Number of tested isolates

#### Campylobacter spp.

All *Campylobacter* isolates found in the tested eggs displayed complete resistance to cefotaxime and nalidixic acid at 100% (95% CI: 64–100) and 100% (95% CI: 92.3–100), respectively (Table 6). However, significant resistance was observed for antibiotics such as chloramphenicol at 77.5% (95% CI: 6.7–100), tetracycline at 63.1% (95% CI: 0.0–100), erythromycin at 66.3% (95% CI: 0.0–100), and ciprofloxacin at 57% (95% CI: 0.0–100). Conversely, less than 50% of *Campylobacter* spp. isolates exhibited resistance to the sulfamethoxazole/trimethoprim combination at 43.6% (95% CI: 28.3–59.5).

**Table 6:** Antibiotic resistance of *Campylobacter* spp. isolates.

Family	Antibiotic	Prevalence%	95% CI	I^2^	N	n	Number of studies
Beta-lactams	Cefotaxime	100	95.6–100	-	39	39	1
Cyclins	Tetracycline	63.1	0.0–100	98.6	64	27	2
Macrolides	Erythromycin	66.3	0.0–100	98.4	64	29	2
Multidrug resistance	Gentamicin/Ciprofloxacin	2.4	0.0–9.9	-	42	1	1
	Nalidixic acid/Ciprofloxacin	2.4	0.0–9.9	-	42	1	1
	Norfloxacin/Ciprofloxacin	2.4	0.0–9.9	-	42	1	1
	Norfloxacin/Ciprofloxacin/Ofloxacin	2.2	0.0–9.9	-	42	1	1
	Tetracycline/Ciprofloxacin	4.8	0.1–13.8	-	42	2	1
	Tetracycline/Nalidixic acid/Norfloxacin/Ofloxacin/Ciprofloxacin	21.4	10.2–35.3	-	42	9	1
Phenicols	Chloramphenicol	77.5	6.7–100	97.2	61	37	2
Quinolones	Ciprofloxacin	56.9	0.0–100	98.9	64	24	2
	Nalidixic acid	100	92.3–100	-	22	22	1
Sulfamides	Sulfamethoxazole/Trimethoprim	43.6	28.3–59.5	-	39	17	1

CI=Confidence Interval, I^2^=Heterogeneity, n=Number of resistant isolates, N=Number of tested isolates

## DISCUSSION

In the present study, a systematic review followed by meta-analysis was conducted to assess the global contamination of chicken eggs intended for human consumption by different bacteria and their antibiotic resistance. The results demonstrate that several harmful microorganisms can contaminate the chicken eggs intended for human consumption and potentially cause foodborne illnesses. The most frequently encountered pathogenic bacteria in eggs belong to the Enterobacteriaceae family, including *Salmonella* spp. and *E. coli*, followed by *Campylobacteraceae* (*Campylobacter* spp.), *Listeriaceae* (*Listeria* spp.), and *Staphylococcaceae* (*S. aureus*). These findings align with those of Adeboye *et al*. [21], who reported that the primary group of bacteria isolated from table eggs were *Enterobacteriaceae*, such as *Salmonella*, which can infect both human and animal communities and are responsible for foodborne illnesses, with clinical manifestations ranging from simple gastroenteritis to septicemia [22]. According to the European Union One Health 2022 Zoonoses report [23], eggs and egg products remain the most commonly food-associated foods with *Salmonella*, particularly Enteritidis serovar, and cause the highest number of foodborne outbreaks. Contamination of poultry by *Salmonella* can occur through two main routes: Vertical transmission (trans-ovarian), which occurs through the reproductive organs, and horizontal transmission, which involves the introduction of bacteria into the egg through the shell [24]. This study ranked *Salmonella* as the most frequently identified bacterium, with a prevalence of 7.1%. This proportion is higher than the results obtained for eggs and egg products, which were 3.1% [25]. The *Salmonella* serovars Enteritidis, Typhimurium, Infantis, and Mbandaka emerged as the most commonly detected serovars in this study, based on the frequency of their reported presence in previous studies. The prevalent *Salmonella* serotypes found in eggs were Heidelberg, Braenderup, Enteritidis, Kentucky, Thompson, and Mbandaka, showing some divergence from those identified in this investigation [26]. These disparities may be attributed to the diversity of analytical techniques employed for bacteria and serotype detection, the number of samples considered, and varied climatic, breeding systems, and geographical conditions.

It is important to note that the intestinal flora of poultry also harbors other significant bacteria, such as *E. coli* [27]. The contamination of eggs by these bacteria tends to increase in shell damage, inadequate cleaning, and suboptimal storage conditions. A high *E. coli* prevalence in eggs can serve as an indicator of poor hygiene practices [28, 29]. The present investigation revealed a general prevalence of *E. coli* in eggs at 18.5%, which could be explained by the conditions of breeding, storage, transport, and/or cross-contamination resulting from product handling, with variations observed from one region to another [30]. It is also crucial to consider other significant foodborne pathogens, among which *Campylobacter* spp. are considered the most common cause of human gastroenteritis worldwide. The consumption of chicken meat and its products is a significant risk factor for human contamination. The findings of the current study revealed a prevalence of 8.0% for *Campylobacter* spp. in eggs, whereas Zbrun *et al*. [31] reported a prevalence of 4%. Similarly, a study conducted in Iran [32] observed a prevalence of 9.9%, which was also lower than that found in this study. This variability can be attributed to disparities in the sensitivity of detection methodologies, approaches to sample collection, egg production environments, and diverse poultry farming systems across different geographic regions. According to the findings reported by the European Food Safety Authority (EFSA) and the European Center for Disease Prevention and Control, *Campylobacter* spp. were frequently examined in broilers and accounted for 43% of the test results [23]. *S. aureus* is one of the most common bacteria found in food, and its ingestion annually causes illness in approximately 241,000 people in the United States and 100,000 people in Europe, according to the EFSA dashboard in 2022. These bacteria are frequently isolated from various foods, including egg products [33, 34]. This high prevalence may be attributed to eggshell contamination during or after laying due to contact with contaminated surfaces. According to some studies, bacteria present on the egg surface can penetrate the shell and infect the egg contents after laying [35]. Furthermore, improper storage conditions and a lack of adherence to hygiene practices can explain the high contamination rate [34]. The presence of *Listeria* spp. in food is also an indicator of poor hygiene. In addition, *L. monocytogenes* causes septicemia and meningitis, which are the most common forms of illness. Moreover, they can cause miscarriages and stillbirths in pregnant women. The presence of these bacteria in eggs is most likely due to contamination from shells during breaking or from the environment [36].

Concerning the two matrices used to determine the prevalence of various bacteria, a higher percentage of bacteria was observed on the eggshell compared to the egg content. This result holds for isolates of *Salmonella* spp., *E. coli*, *Campylobacter* spp., *S. aureus*, *P. mirabilis*, and *Klebsiella* spp., for which higher detection rates were observed on the eggshell than in the egg content. This observation is likely due to exposure to poor sanitary conditions in egg-laying farms, environmental conditions in different regions of egg production, housing systems, or egg transportation, storage, and packaging systems [9]. Ordinarily, eggs possess a natural defense system against microbes consisting of the cuticle, calcium-rich shell, and shell membrane. The egg, which contains several proteins including lysozyme, also exhibits antimicrobial properties [37]. However, the study by Favier *et al*. [38] identified three bacteria with higher prevalence in egg content than on eggshells. These bacteria are *Listeria* spp., *Enterobacter* spp., and *Yersinia* spp. In addition to horizontal and transovarian contamination, egg content can become contaminated through the ingress of microorganisms through the cracked eggshell. Therefore, the bacteria present on the eggshell may be identical to those found within the egg content, leading to contamination [39]. In addition, prolonged storage of eggs under unsuitable conditions can weaken their protective barriers, thereby permeating the egg contents with various contaminants. The presence of these bacteria indicates poor hygiene practices and likely arises from contact with other previously contaminated animal products [8]. Uniformly, the presence of *Citrobacter* spp. in eggs is likely the result of environmental contamination (such as litter or feces), highlighting the significance of this factor in the contamination of the eggs studied.

Antibiotics have been extensively used in farming systems for decades to improve production and manage infections in both animals and humans [40]. Studies across different countries have observed significant variations in the use of these antimicrobials in poultry for therapeutic purposes. While some studies suggest that the use of antibiotics as growth promoters is commonplace in certain parts of the world, others highlight the use of banned antibiotics, such as the continued use of nitrofurans in some Nigerian farms [41]. A study by Malijan *et al*. [42] conducted in Southeast Asia identified multiple classes of antibiotics used in poultry, including aminoglycosides, penicillins, colistin, cotrimoxazole, macrolides, quinolones, and tetracyclines. Some of these antibiotics are designated for specific infections or as a last resort. In large-scale poultry farms, antibiotics are often administered for optimal and cost-effective results by incorporating them into feed or drinking water. Injection is another viable method although injection may lead to a build-up of the drug in tissues, delaying its elimination [43]. The misuse of antibiotics contributes to the presence of egg resi-dues. Several antibiotic residues were found in table eggs from various countries, including Bangladesh, China, Egypt, Nigeria, and Tanzania, with predominant antibiotics such as amoxicillin, quinolones, tetracyclines, sulfonamides, beta-lactams, cephalosporins, and chloramphenicol [44]. These residues deposited in egg compartments pose significant health risks to consumers [45]. The observed results indicate a high level of resistance among *Salmonella* isolates to nitrofurantoin, novobiocin, and polymyxin, with resistance rates reaching 100% in certain cases. However, these findings are based on only two studies [46–51]. on each antibiotic, which may limit the generalizability of the results. In addition, significant variations in antibiotic resistance have been observed for *Salmonella* in different countries. The antibiotics ampicillin, gentamicin, and streptomycin are widely distributed in Africa, Asia, Europe, America, and Oceania. The variabilities observed for *Salmonella* antibiotic resistance depend on several factors, including serovars, farm, period, and specific antimicrobial agents [46]. According to the present findings by Abubakar and Salman [52], gentamicin, a widely used and affordable antibiotic for treating infections in humans and poultry, exhibits higher resistance levels in Africa and Europe. Conversely, some *Salmonella* isolates from Asian countries remain susceptible to streptomycin, whereas those from African and European countries show high resistance. For example, in Russia, >90% of *Salmonella* isolates are streptomycin-resistant. This is likely due to the common use of streptomycin as a growth promoter in animals, which facilitates the transfer of resistance to the food chain [53]. This meta-analysis provides a comprehensive overview of the resistance of *E. coli* isolates to various antibiotics, revealing significant insights into the prevalence and variability of resistance across different antibiotic classes. Complete resistance was observed for penicillin G and cephalosporins such as cephalexin and cefuroxime. Amoxicillin showed a 72.2% resistance rate across three studies, while amoxicillin-clavulanic acid exhibited 100% resistance in a single study. The high resistance to β-lactams is largely attributed to the production of extended-spectrum β-lactamases (ESBLs), which degrade these antibiotics. These enzymes can hydrolyze penicillin, cephalosporins, and monobactams, rendering these antibiotics ineffective. ESBLs are not active against carbapenems. ESBL activity can be inhibited by β-lactamase inhibitors [54]. Resistance to other classes of antibiotics, such as macrolides, nitrofurans, quinolones, and tetracyclines, was reported for *E. coli* in this study. The use of nitrofurans has been prohibited because of their genotoxic and carcinogenic effects. Quinolones are considered first-line antibiotics for treating *E. coli* infections in poultry, including respiratory, genitourinary tract, and gastroenteric infections. Cyclines and macrolides are the most frequently used antimicrobial agents in veterinary medicine for treating domestic animals, including poultry. The resistance of *E. coli* isolates found in eggs underscores the need for legal alternative antimicrobial options to improve egg production. *Campylobacter* exhibits complete resistance to antibiotics in the quinolone family, such as nalidixic acid and β-lactam third-generation cephalosporins (cefotaxime). However, other research suggests that β-lactam antibiotics generally have limited effectiveness against *Campylobacter*. This resistance appears to arise from both intrinsic resistance mechanisms within bacteria and the production of β-lactamase enzymes [55]. A small-scale study conducted in Kenya [56] documented a high prevalence of resistance among *Campylobacter* isolates from poultry farms to nalidixic acid (77.4%) and other antibiotics. Furthermore, this study observed a similarity between the antibiotic resistance profiles of bacteria isolated from both eggs and poultry, suggesting a potential route of vertical transmission from parent birds to eggs.

Antimicrobial resistance is a significant global public health concern, and resistant bacterial strains pose a serious threat to healthcare communities [57]. In this review, two studies reported multidrug resistance in *E. coli*, with 10 distinct resistance patterns identified. In addition, one study identified five resistance patterns of *Campylobacter*. The excessive use of antimicrobial agents may contribute to resistance, particularly in intensive egg production systems. These systems often involve limited space and poor conditions, such as inadequate nutrition, hygiene, and feeding practices, which increase the risk of disease transmission and necessitate antimicrobial use, potentially driving the emergence of resistant strains [58].

## CONCLUSION

This systematic review and meta-analysis highlight a significant global prevalence of bacterial contamination in table eggs, predominantly involving Salmonella spp., *E. coli*, *S. aureus*, Campylobacter spp., and *L. monocytogenes*. The study notably identifies higher contamination rates on eggshells compared to egg contents. Furthermore, a concerning trend of antibiotic resistance was observed, with *Salmonella* spp. and *E. coli* isolates exhibiting extensive resistance to widely used antibiotics, including complete resistance (100%) to nitrofurantoin, novobiocin, polymyxin, and penicillin G. Regional variations were pronounced, particularly with elevated resistance levels in isolates from Asia and Africa, highlighting significant public health implications.

The robust methodological approach, employing advanced bibliometric analyses and comprehensive database searches spanning over four decades (1979–2022), ensures extensive coverage and reli-ability of the findings. The use of rigorous statistical analysis, including heterogeneity assessment and subgroup analyses, further enhances the validity and generalizability of the results.

Despite the methodological rigor, the study faced limitations, including variability in antibiotic resistance interpretation criteria across studies and limited data availability for certain antibiotics, potentially affecting generalizability. In addition, inherent methodological variations among included studies may have introduced heterogeneity in prevalence estimates.

Future research should focus on standardized methodological frameworks for detecting bacterial contamination and antibiotic resistance in table eggs globally. Longitudinal studies assessing the impact of biosecurity interventions and antimicrobial stewardship programs on reducing bacterial prevalence and antibiotic resistance would provide valuable insights. Furthermore, comprehensive genomic studies are warranted to elucidate resistance mechanisms, thereby informing more effective, targeted strategies for mitigating antibiotic resistance risks in poultry production and improving global food safety.

## DATA AVAILABILITY

The supplementary data can be obtained from the corresponding author upon reasonable request.

## AUTHORS’ CONTRIBUTIONS

CH, PS, and AT: Conceptualized the study, methodology, and supervised the manuscript. CH, PS, PA, GD, NK, and AT: Formal analysis. CH and AT: Data curation. CH: Writing – original draft preparation. CH, GD, NK, PA, AT, and PS: Writing – review and editing. AT: Visualized the study. All authors have read and agreed to the publication of the final version of the manuscript.
